# Ocean Acidification, Iodine Bioavailability, and Cardiovascular Health: A Review of Possible Emerging Risks

**DOI:** 10.3390/jcdd12110418

**Published:** 2025-10-22

**Authors:** Charalampos Milionis, Costas Thomopoulos, Emilia Papakonstantinou, Ioannis Ilias

**Affiliations:** 1 Department of Endocrinology, Diabetes & Metabolism, Elena Venizelou Hospital, 11521 Athens, Greece; pesscharis@hotmail.com; 2 Department of Cardiology, Laikon General Hospital, 11527 Athens, Greece; thokos@otenet.gr; 3 Laboratory of Dietetics and Quality of Life, Department of Food Science and Human Nutrition, School of Food and Nutritional Sciences, Agricultural University of Athens, 11855 Athens, Greece; emiliap@aua.gr; 4 Department of Endocrinology, Hippokration General Hospital, 11527 Athens, Greece

**Keywords:** climate change, iodine, nutrition, cardiovascular disease

## Abstract

Anthropogenic climate change drives ocean acidification, which alters marine iodine cycling and increases bioaccumulation in marine ecosystems. This environmental shift may alter marine iodine cycling and, under certain conditions, lead to increased dietary and atmospheric iodine exposure, particularly in coastal populations, with potential risks for thyroid dysfunction and downstream cardiovascular complications. Experimental data suggest that acidification may enhance iodine uptake in marine organisms such as kelp and seafood, with possible implications for consumption by humans. Because chronic iodine excess has already been associated with thyroid disease and its related cardiovascular disorders, these connections are worthy of further examination. In this narrative review we provide a synthesis of the possible mechanistic pathways by which ocean acidification, iodine bioavailability, thyroid function, and cardiovascular health may be connected. We also highlight the need for ongoing investigation, environmental monitoring, and interdisciplinary collaboration to further explain and address these tentative associations.

## 1. Climate Change, Ocean Acidification, and the Global Iodine Cycle

Global warming and its associated climate changes are projected to profoundly influence the global iodine supply, with downstream effects on marine biogeochemistry, atmospheric chemistry, and human nutrition [1]. Rising atmospheric carbon dioxide (CO_2_), the principal driver of contemporary climate change, readily dissolves in seawater where it forms carbonic acid (H_2_CO_3_). The dissociation of this weak acid increases hydrogen ion concentrations, thereby lowering ocean alkalinity. This progressive ocean acidification is expected to interact with iodine speciation and sea-air fluxes through indirect biological and redox pathways that also depend on mixing and light [2,3], altering the iodine content of marine organisms that constitute key dietary sources of this micronutrient. Consequently, the marine iodine cycle stands at the nexus of climate-driven environmental change and human health. In this narrative review we focus on the relationship between ocean acidification, iodine bioavailability, and cardiovascular health. To this end we searched PubMed & ScienceDirect up to April 2025 with the search terms “(Ocean AND Acidification) OR (climate AND change) OR (global AND warming) OR (climate AND change) AND iodine AND human”. The search produced 476 articles. The abstracts were examined and 66 papers were further retained to be examined in full. A few more articles were selected from the lists of references in the selected literature.

### 1.1. Climate Change, Ozone, and Marine Iodine Cycling

Increased tropospheric ozone (O_3_), resulting from anthropogenic emissions, reacts with marine iodide (I^−^) to release volatile gaseous iodine (I_2_) into the atmosphere [4]. This reaction participates in a negative feedback loop that reduces ozone concentrations in the lower atmosphere [5]. Associations between warmer surface conditions and elevated iodide reflect indirect physical-biochemical controls (e.g., vertical mixing, light, and primary production) rather than a direct temperature effect. Consequently, iodide may increase in some regions while remaining unchanged or decreasing elsewhere [2,3]. Observational studies of Alpine and Greenland ice cores have revealed a threefold increase in atmospheric iodine deposition between 1950 and 2010, attributed largely to enhanced anthropogenic O_3_ production and subsequent oceanic emissions [6]. This acceleration is expected to persist into the 21st century [6]. Melting polar ice caps contribute by exposing frozen phytoplankton to increased solar radiation, releasing stored iodide that reacts with O_3_ to form volatile iodines; thinner, refrozen ice layers further facilitate iodine release [7]. Climate change, including sea ice retreat and O_3_-driven emissions, is therefore projected to markedly increase biogenic gaseous iodine emissions [8]. These dynamics highlight the tightly coupled interplay between atmospheric chemistry, ocean circulation, and iodine cycling. Regionally varying surface ocean iodide arises from a combination of physical mixing and biological uptake/regeneration processes, which also condition sea-air iodine fluxes [2,3].

### 1.2. Ocean Acidification and Iodine Speciation

Ocean acidification is hypothesized to influence iodine speciation in the surface ocean, potentially shifting the I^-^/IO3^−^ balance via changes in biology and redox cycling the sign and magnitude of such shifts remain uncertain across regions [2,3]. Ocean acidification has been shown to stimulate the growth of certain seaweed species and increase their iodide accumulation [8], raising concerns about excessive iodine intake in populations with seaweed-rich diets [9]. Experimental cultivation of Japanese kelp (Saccharina japonica) under acidified conditions (pH 7.8) demonstrated a 40% increase in iodine content compared to controls [10]. More in detail, in the laboratory or mesocosm (controlled, experimental ecological systems) experiments, elevated pCO_2_ increased iodine accumulation in seaweeds (*p* < 0.0001) with slower decline over time, and abalone fed S. japonica grown and reared under elevated pCO_2_ showed the greatest accumulation (*p* < 0.0001) [10]. These mesocosm and cultivation findings do not, by themselves, establish basin-scale increases in seawater iodine; surface ocean I^−^ is constrained by speciation and mixing processes that vary by region [2,3]. Atlantic cod (Gadus morhua) exhibited a 20% rise in muscle iodine under similar low-pH conditions [11]. Mechanistically, these changes may be driven by down-regulation of vanadium-dependent haloperoxidase genes in kelps, reducing oxidative stress and favoring iodine retention within algal tissues, with downstream propagation through food webs, as evidenced by abalone consuming iodine-enriched diets [10]. Notably, such dietary enrichment altered thyroid hormone synthesis in abalone, suggesting that ecosystem-level iodine perturbations may have endocrine implications for marine consumers and potentially for humans [10]. Sea surface iodide concentrations follow a tropical water maximum global pattern, and sea surface temperature is the dominant parameter controlling both iodine speciation and the iodide-to-iodate ratio in the oceans’ surface [2,3]. These occur, however, against the background of climate-related stressors such as ocean warming, acidification, deoxygenation, and pollution, which interact to influence marine productivity, community structure, and ultimately the pathways of oceanic iodine cycling [12,13,14,15].

### 1.3. Microbial Processes, Nitrification, and Iodine Fluxes

Ocean acidification also interferes with microbial nitrification, a process that oxidizes I^−^ back to IO3^−^ [16]. Reduced nitrification, a 3–44% decline in ammonia oxidation with only a 0.1 unit decrease in pH, elevate surface iodide concentrations [16]. Modeling studies predict a near-linear global rise of 3.6% in iodine emissions, with regional increases up to 7–10% in subtropical gyres (weather systems with high barometric pressure at their centers situated beneath regions of subtropical high pressure) [4,17]. These iodine radicals catalytically destroy ozone, thereby shaping coastal air quality and potentially contributing to feedback in climate regulation.

### 1.4. Regional and Ecological Variability

Despite these broad patterns, the net effect of climate change on iodine remains complex and context-specific. While acidification may promote iodine retention in seaweeds, concurrent warming threatens kelp forests in several regions. For instance, giant kelp populations off Tasmania have collapsed due to warming waters, removing a major marine iodine source [18]. Such region- and species-specific responses complicate risk assessments and predictive modeling [4,10]. Compounding this complexity, iodine concentrations in seaweeds vary not only by species but also by harvest season and processing methods, further challenging exposure estimates. Of note, a recent study warns that global climate change, through rising ocean temperatures exceeding 28 °C, could halve populations of the abundant phytoplankton Prochlorococcus by the end of the century [19], severely disrupting marine food webs, biodiversity, and planetary oxygen production (this cyanobacterium forms the base of tropical and subtropical ocean ecosystems). Prochlorococcus does not inherently contain significant amounts of iodine within its cells, but rather it produces iodine-containing compounds like methyl iodide (CH_3_I)[20]. This organoiodine compound is a gaseous trace gas that can be released into the atmosphere, contributing to atmospheric chemistry [20]. Thus, climate change may diminish the volatile iodine emissions from declining phytoplankton like Prochlorococcus.

### 1.5. Deposition Pathways, Agricultural, Animal and Human Impact

Iodine is an essential micronutrient required for thyroid hormone synthesis, with a recommended daily intake of 150 μg for adults and an upper tolerable limit of 1100 μg [21]. Seaweeds (100–2000 μg/g), fish (50–200 μg/100 g), and shellfish (100–300 μg/100 g) [22] remain dominant dietary sources in high-consuming regions such as Japan, Iceland, and parts of Southeast Asia—often resulting in intakes surpassing 1000 μg/day [23]. However, for most populations worldwide, dairy products and iodized salt constitute the primary sources, contributing to 50–70% of its intake [24,25]. The iodine content of marine organisms is influenced by species, trophic position, diet, and environmental conditions. For instance, Gadus morhua (Atlantic cod), an omnivorous species, does not accumulate iodine directly from macroalgae [26], and while oysters, shellfish, and white fish such as haddock and pollock are iodine-rich, fatty fish like salmon contain considerably lower levels [27,28,29]. Moreover, iodine losses during trophic transfer and cooking can reduce actual dietary exposure, meaning estimates based solely on algal bioaccumulation may overstate intake. Climate-driven changes further complicate these dynamics: although ocean acidification may have negligible effects on iodine availability for mixed-diet populations [30], atmospheric iodine deposition onto soils and crops [8], seasonal variability in milk iodine concentrations [31], and the expansion of iodine-enriched inundated land due to melting ice caps [32] may alter terrestrial food iodine content. While the contribution of inhaled iodine to total intake is not known [33], it is conceivable that exposure due to sea-spray aerosols or anthropogenic sources— such as seaweed fertilizer and disinfectants—could, under some conditions, result in excess exposure to iodine and possibly influence thyroid function [33,34].

### 1.6. Emerging Implications for Human Health

Overall, the combined effects of global warming, ozone interactions, sea ice retreat, and ocean acidification may reshape the marine iodine cycle. The increased release of volatile iodine into the atmosphere and its deposition onto land, coupled with enhanced iodine accumulation in seaweeds and marine organisms, can create a more iodine-rich biosphere. While this may reduce the risk of iodine deficiency disorders in some populations, it may simultaneously raise the threat of chronic iodine excess in others. The absence of long-term human studies directly linking acidification-driven iodine changes with health outcomes underscores the need for ongoing monitoring of dietary iodine intake and comprehensive assessment of climate-driven nutritional transitions [10,35,36].

## 2. Mechanisms and Prevalence of Thyroid Dysfunction

Iodine metabolism is essential for normal thyroid function and overall metabolic balance. After iodine is ingested, it is absorbed in the gastrointestinal tract and transported through the bloodstream to the thyroid gland. The thyroid follicular cells actively trap iodine through a sodium-iodide (NIS) symporter at their basolateral membrane. Low iodine levels increase the quantity of NIS and stimulate its uptake [37,38]. In contrast, high iodine levels suppress NIS expression and lower its uptake. Pendrin is another iodine transporter, which is located on the apical surface of thyrocytes and mediates iodine efflux into the thyroid follicular lumen [37,38]. Inside the thyroid gland, iodide is oxidized in an organification reaction that involves thyroid peroxidase (TPO) and hydrogen peroxide. Afterward, the reactive iodine is bound to tyrosine residues on thyroglobulin (Tg), a protein stored in the colloid. This process forms monoiodotyrosine (MIT) and diiodotyrosine (DIT). TPO further catalyzes the coupling of these molecules to form either T3 or T4, depending on the number of iodine atoms in the iodotyrosines [37,38]. After coupling, Tg is taken back into the thyrocyte, where it is processed in lysosomes to release T3 and T4. The latter are then secreted into the bloodstream. The hypothalamic-pituitary-thyroid axis tightly regulates the production of thyroid hormones [38]. Epidemiological data show a U-shaped relationship between iodine intake and thyroid outcomes, meaning both low and high iodine exposure are associated with thyroid abnormalities, but of different types [39]. Low iodine intake primarily increases the risk of goiter and hypothyroidism [40], while excess iodine can trigger or worsen thyroid autoimmunity, leading to hyperthyroidism or hypothyroidism, particularly in genetically predisposed individuals [41,42,43]. Epidemiological studies from Japan indicate that hypothyroidism is less prevalent in coastal regions, where seaweed consumption contributes to high iodine intake, compared to inland areas. Reported prevalence rates in coastal populations range from 0–9.7% [43], with one study documenting only 1.3% [44]. In contrast, comparative analyses demonstrate significantly higher rates of both subclinical (16.2% vs. 6.9%) and clinical hypothyroidism (1.3% vs. 0.3%) in inland versus coastal populations [44]. Although excessive iodine intake, largely from seaweed, is recognized as a potential cause of hypothyroidism, the prevalence in coastal Japan is not higher than in other iodine-sufficient regions [43], likely reflecting an adaptive “escape phenomenon” that mitigates the inhibitory effects of iodine excess on thyroid hormone synthesis.

Chronic iodine excess disrupts thyroid homeostasis through several mechanisms:

[a]. WolffChaikoff Effect: High iodine levels inhibit thyroid hormone synthesis by downregulating NIS symporter activity and TPO, causing transient hypothyroidism [45]. This is an effective means of dismissing the excessive quantities of iodine and thus preventing the thyroid from synthesizing immoderate amounts of thyroid hormones. The acute phase of this phenomenon lasts for a few days, and subsequently, the organification of intrathyroidal iodide resumes, restoring normal T4 and T3 synthesis. However, in susceptible individuals, including those with pre-existing thyroid conditions, the thyroid gland may fail to escape the WolffChaikoff effect. This can result in a persistent inhibition of thyroid hormone synthesis, leading to clinical hypothyroidism [46,47].
[b]. Jod-Basedow Effect: In iodine-replete individuals, excess iodine can trigger autonomous thyroid hormone production, particularly in those with nodular goiter or latent Graves’ disease, resulting in hyperthyroidism [47]. This is a physiological failure of the thyroid gland, in which patients develop hyperthyroidism by escaping the physiological negative feedback response to the surplus of iodine. If left unrecognized, it could lead to serious consequences, such as arrhythmias, heart failure, pulmonary arterial hypertension, cerebrovascular and pulmonary embolism, and cardiomyopathy [48].
[c]. Autoimmune Thyroiditis: Chronic iodine excess may exacerbate autoimmune thyroid diseases, such as Hashimoto’s thyroiditis, by promoting oxidative stress and immune activation [49]. Autoimmune thyroiditis begins with the accumulation of macrophages, dendritic cells, and plasma cells within the thyroid tissue. This initial infiltration triggers a dysregulated immune response marked by a shift toward specific T-helper subsets and reduced activity of regulatory T cells, leading to increased production of inflammatory cytokines. The resulting immune imbalance activates pyroptotic and apoptotic pathways, ultimately causing targeted destruction of thyroid follicular cells by cytotoxic T lymphocytes and natural killer cells [50]. Excessive iodine intake may promote thyroid autoimmunity through several pathways. First, it increases the iodination of thyroglobulin, enhancing its immunogenicity by creating new epitopes. Second, it increases the production of reactive oxygen species, which upregulate adhesion molecules on thyrocytes, and it promotes their apoptosis. Third, it amplifies pro-inflammatory responses [51].

Exposure to excessive iodine is an environmental factor that can disturb the thyroid’s functional integrity. Abnormalities in the function of the thyroid gland can manifest as a deficiency or excess of thyroid hormones. Hypothyroidism is characterized by elevated thyroid-stimulating hormone (TSH) levels and reduced T4 and T3 levels, whereas hyperthyroidism features suppressed TSH levels and elevated T4 and T3 levels. Both conditions are more prevalent in areas with high iodine intake. For example, in coastal Japan, the prevalence of hypothyroidism is 5–7%, and hyperthyroidism affects 2–3% of adults, compared to global averages of 3–5% and 1–2%, respectively [52]. Similar patterns are observed in Iceland and coastal China, where marine diets predominate [22]. Although direct epidemiological data are lacking, alterations in marine iodine bioavailability due to ocean acidification may, through the thyroid–cardiovascular axis, influence cardiovascular risk (see next section). Elevated iodine intake in coastal populations reliant on marine foods could predispose to thyroid dysfunction, which is linked to dyslipidemia, atherosclerosis, arrhythmias, and heart failure. While causality remains unproven, this potential pathway highlights a plausible environmental contribution to cardiovascular disease that warrants further investigation.

## 3. Thyroid Dysfunction and Cardiovascular Complications

The increased or reduced action of thyroid hormones on specific molecular pathways in the heart and vasculature leads to relevant cardiovascular derangements. Thyroid hormones regulate cardiovascular function through both genomic and non-genomic pathways, influencing heart rate, contractility, and vascular tone [53]. Non-genomic effects influence primarily the transport of amino acids, sugars, and calcium across the cell membrane. Nuclear effects are mediated by the binding of thyroid hormone to specific receptor proteins in the nucleus. This results in a significant impact on the contractile apparatus and the sarcoplasmic reticulum of the cardiomyocytes [54]. Thyroid dysfunction disrupts these processes, leading to distinct cardiovascular complications. While some studies suggest potential links between high water iodine and carotid artery diameter [55], robust epidemiological evidence directly linking iodine exposure to cardiovascular outcomes is limited compared to the established relationship with thyroid disorders [56].

### 3.1. Overt Hypothyroidism

Hypothyroidism promotes dyslipidemia (elevated low-density lipoprotein cholesterol and triglycerides), endothelial dysfunction, and arterial stiffness, accelerating atherosclerosis [57]. It also reduces cardiac output and increases systemic vascular resistance, thereby predisposing the heart to failure. A meta-analysis of cohort studies reported a 1.7-fold increased risk of heart failure in patients with subclinical hypothyroidism (TSH > 4.5 mlU/L) [58]. Hypothyroidism is also linked to diastolic hypertension and increased cardiovascular mortality [59].

### 3.2. Overt Hyperthyroidism

Hyperthyroidism increases cardiac output, heart rate, and myocardial oxygen demand, leading to tachyarrhythmias, particularly atrial fibrillation (10–15% prevalence in hyperthyroid patients) [60]. Atrial fibrillation, in turn, elevates the risk of thromboembolism and stroke. Hyperthyroidism also promotes oxidative stress and inflammation, thereby accelerating the progression of atherosclerosis [61]. Severe cases may lead to high-output heart failure, especially in elderly patients [62].

### 3.3. Subclinical Thyroid Dysfunction

It is not only the overt forms of hypothyroidism or hyperthyroidism that are detrimental to cardiovascular integrity [63]. Subclinical thyroid dysfunction, characterized by abnormal TSH with normal T4 and T3 levels, also carries cardiovascular risks. Subclinical hypothyroidism is associated with a 1.5-fold increased risk of coronary artery disease, while subclinical hyperthyroidism doubles the risk of atrial fibrillation [64,65].

## 4. Global Health Perspectives: Vulnerable Populations

Coastal populations, particularly in low- and middle-income countries, are disproportionately affected by rising iodine exposure. In regions such as Southeast Asia, where seaweed is a dietary staple, the average iodine intake often exceeds 1500 μg per day, far above the tolerable limit [65]. Small island nations, such as those in the Maldives and Seychelles, face similar risks due to their reliance on marine resources. Socioeconomic factors, including limited access to healthcare and nutritional education, exacerbate vulnerability in these communities [66]. Indigenous coastal populations, such as the Inuit in Arctic regions, also face unique challenges. Their traditional diets, rich in marine mammals and fish, provide iodine levels up to 2000 μg/day, thus increasing the risk of thyroid dysfunction as acidification amplifies marine iodine content [67]. Gender disparities further complicate the issue, since women are more susceptible to iodine-induced thyroid disorders due to hormonal influences and higher autoimmune thyroid disease prevalence [68]. Urban coastal populations in developed nations, such as those in the United States and Europe, are not immune to these issues. The growing popularity of seaweed-based foods and supplements has increased iodine intake, particularly among health-conscious consumers [69]. As ocean acidification progresses, these populations may face rising rates of thyroid and cardiovascular morbidity.

## 5. Clinical Management and Preventive Strategies vis-à-vis Ocean Acidification and Iodine

While iodine deficiency is widely recognized as a health risk, the consequences of iodine’s excess are less well understood. The surplus of iodine has been implicated in a variety of adverse outcomes on thyroid function and systemic health. Overconsumption of iodine can trigger autoimmune thyroid conditions through the polarization of immune cells and alterations in gut microbiota. Moreover, excess iodine can be associated with an increased risk of cardiovascular diseases due to oxidative stress, inflammation, and endothelial dysfunction. It may also play a role in the development of papillary thyroid carcinoma by causing genetic mutations and enhancing cancer cell proliferation [70]. In addition, it has been linked to neurotoxic effects, impairing learning and memory, compromising the development of the neonatal brain, and possibly favoring the progression of neurodegenerative diseases. Lastly, it may contribute to renal impairment in susceptible groups [71].

Addressing the health impact of iodine excess requires a multifaceted approach, integrating clinical, public health, and environmental strategies:

[a]. Screening and Monitoring: Routine thyroid function testing (TSH, free T4, free T3) should be prioritized in high-risk populations, including coastal residents, pregnant women, and individuals with cardiovascular risk factors. Annual screening is recommended for those with dietary iodine intake exceeding 500 μg/day [72]. Thyroid ultrasound and anti-thyroid antibody testing can identify early autoimmune changes. Timely detection enables appropriate intervention, reducing the risk of progression to clinically overt thyroid dysfunction.
[b]. Dietary Interventions: Public health campaigns should promote a balanced intake of iodine, emphasizing moderation in the consumption of seaweed and seafood. Iodized salt, providing 100–200 μg/day, can maintain sufficiency without excess [73]. Nutritional counseling should be tailored to the cultural dietary practices of coastal communities. The interaction with local leaders and nutritionists can help align messaging with community norms and improve adherence.
[c]. Environmental Surveillance: Real-time assessment of marine iodine levels, using advanced spectrometry and remote sensing, could ideally inform public health policies. However, the measurement of seawater iodide and iodate concentrations using such methods is not yet possible. For now, these compounds are measured in discrete water samples collected during research cruises and field campaigns [74]. International collaboration is needed to monitor the trend of ocean acidification and its impact on health [75]. Incorporating environmental data into health surveillance systems can strengthen predictive models for iodine-related thyroid risks.
[d]. Policy and Advocacy: Reducing CO2 emissions through global agreements such as the Paris Accord is essential to mitigate ocean acidification and its effects on marine iodine cycling [76]. Local measures, including regulation of seaweed-based supplements, further help limit excessive iodine exposure. Regulatory thresholds are critical [77]: the European Food Safety Authority set the upper tolerable intake at 600 µg/day in adults, and EU regulations cap iodine in dried seaweed at 20 mg/kg dry weight [78]. Australia and New Zealand impose stricter limits (<1000 mg/kg) on imported seaweed, with batch testing to reduce risks from overexposure [79]. Monitoring of iodized salt remains vital to balance deficiency prevention with risks of excess, particularly alongside salt reduction policies [80]. In South Africa, mandatory iodization (35–65 mg/kg since 2006) has been evaluated with urinary iodine concentration data, though recent data are limited. The 2016 salt reduction policy raised concerns about adequacy, reinforcing the need for continued monitoring [81]. Coordinated action between health and environmental authorities is essential to integrate monitoring data into effective interventions that minimize iodine-related endocrine risks in vulnerable populations [80].
[e]. Education and Training: Medical curricula should include climate-driven health risks, equipping clinicians to recognize and manage iodine-induced disorders. Community education programs can raise awareness of safe dietary practices [82]. Empowering both health care providers and the public with knowledge is expected to foster prevention and long-term health resilience.

## 6. Implications for International Action and Governance

The interplay between ocean acidification, the iodine cycle, and human health remains significantly underrepresented in global policy frameworks. Anthropogenic carbon emissions are not only warming the planet but also disrupting marine biogeochemical cycles, potentially altering iodine availability in marine ecosystems. Despite the health hazards associated with iodine excess, including thyroid dysfunction and cardiovascular complications, current international agreements do not explicitly address the consequences of ocean acidification on human nutrition and endocrine health.

Several multilateral environmental treaties touch on related issues, albeit indirectly. The United Nations Framework Convention on Climate Change (UNFCCC) and its implementing treaty, the Paris Agreement, aim to limit global warming by reducing carbon emissions [83]. Similarly, the United Nations Convention on the Law of the Sea (UNCLOS) mandates the protection of marine biodiversity and the sustainable use of ocean resources [84]. The 2030 Agenda for Sustainable Development acknowledges that social conditions, human health, and environmental degradation, among others, are intertwined and should be addressed together [85]. Regional initiatives, such as those led by Caribbean and Pacific Island nations, have also called for action against ocean acidification. However, these frameworks do not include provisions for monitoring trace element dynamics or assessing their implications for human populations.

To address these gaps, global governance structures must evolve to incorporate trace element monitoring, evaluate dietary exposure risks, and strengthen health system preparedness. Integrated policy frameworks are needed to bridge marine environmental monitoring with public health planning. These should emphasize the interconnectedness of environmental, animal, and human health and support cross-sectoral strategies for managing iodine-related risks. As the burden of iodine-related health outcomes is unevenly distributed across populations, culturally sensitive interventions and food security strategies are essential to protect vulnerable communities.

## 7. Research Gaps and Future Directions vis-à-vis Ocean Acidification and Iodine

Several critical knowledge gaps hinder the effective management of iodine overexposure because of oceanic acidification. Addressing these issues is essential for informing clinical strategies, environmental policy, and nutritional guidelines:

[a]. Quantifying Exposure: Longitudinal studies are needed to quantify the impact of rising marine iodine levels on human exposure and health outcomes. Current data are limited to small-scale experimental studies [11]. Standardized biomonitoring protocols and regional dietary assessments are needed to establish causal links between marine iodine variability and human health indicators.
[b]. Assessment of Synergistic Stressors: The combined effects of acidification and other climate-driven factors, such as heavy metal bioaccumulation and microplastic pollution, on thyroid health remain underexplored [86]. Future studies should adopt integrated exposure models that reflect real-world, multi-contaminant scenarios in coastal food chains.

Under scenarios of ocean acidification, quantitative estimates of projected iodine exposure can be integrated by coupling biogeochemical ocean models with atmospheric chemistry models. Ocean acidification impacts nitrification rates, which in turn affect the oxidation of iodide to iodate in the ocean surface mixed layer. Studies suggest that acidification-induced decreases in nitrification could lead to enhanced iodide concentrations in seawater, with modeled increases of up to 10% or more in subtropical gyres [4]. This increase in surface ocean iodide then scales with inorganic iodine emissions to the atmosphere, estimated using models such as GEOS-Chem, where a 1% increase in aqueous iodide results in approximately a 0.7% rise in iodine emissions [4]. These emissions have implications for atmospheric chemistry, notably altering ozone destruction cycles and human iodine exposure via marine food webs. Experimental data also shows ocean acidification increases iodine accumulation in kelp, a key dietary source, enhancing iodine availability in coastal food webs [4,10]. Thus, integrating quantitative estimates involves: (1) mechanistic biogeochemical modeling of iodine speciation and cycling influenced by acidification-driven changes in microbial nitrification, (2) coupling these oceanic iodide concentration changes to atmospheric emission models for projected iodine fluxes, and (3) incorporating bioaccumulation effects in marine organisms to assess food chain iodine exposure. Sensitivity analyses around nitrification perturbations and acidification scenarios improve projections for human health risk assessments, highlighting regional heterogeneity in iodine exposure risk linked to oceanographic variability. This integrated approach is critical to reliably predict iodine exposure under evolving ocean acidification conditions and inform public health and environmental policies [4,10].

[c]. Integration of Population Variability: Genetic, demographic, and environmental factors influencing iodine metabolism and thyroid susceptibility require further investigation, particularly in diverse populations [73]. This includes evaluating differential responses to iodine exposure across age groups, sex, nutritional statuses, and genetic backgrounds to inform personalized risk assessments.
[d]. Evaluation of Intervention Efficacy: The design of interventions should consider the iodine kinetics at the population level. Randomized trials are needed to evaluate the effectiveness of dietary interventions and screening programs in reducing thyroid and cardiovascular morbidity.

Emerging technologies (e.g., machine learning models to describe high-risk regions and populations) could enhance targeted interventions. Interdisciplinary research, bridging marine biology, endocrinology, and cardiology, is essential to address this complex issue.

## 8. Conclusions

**Figure 1:**
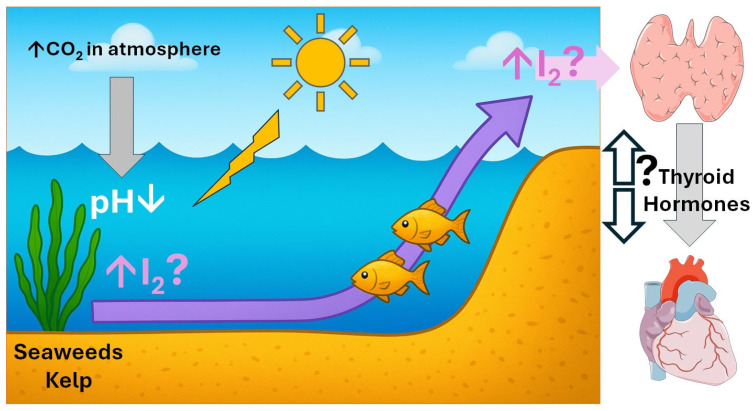
Climate change-related alterations such as global warming, ocean acidification and pollution, may predispose populations with high marine dietary intake to iodine excess (small purple arrows indicate high iodine content, large purple arrow indicates the flow of increased iodine from the marine environment to the thyroid), thereby heightening the risk of thyroid dysfunction. Both hypothyroidism and hyperthyroidism can exert cardiovascular effects. However, no studies have yet demonstrated a direct causal link between ocean acidification and cardiovascular morbidity (question marks denote areas of uncertainty). Figure created with Chat GPT 5 (27 August 2025 version; Large language model, https://chatgpt.com/; accessed on 28 August 2025) and with images provided by Servier Medical Art (https://smart.servier.com/ accessed on 28 August 2025), licensed under CC BY 4.0 (https://creativecommons.org/licenses/by/4.0/ accessed on 25 August 2025).

Ocean acidification, an anthropogenic consequence of climate change, may influence marine iodine cycling, with implications for downstream effects on human thyroid health and cardiovascular health (Figure 1).

Increased marine iodine bioavailability in certain ecological and dietary situations might play a role in altering iodine exposure, particularly in coastal communities depending on marine-derived foods, under some ecological and diet conditions. Identification of iodine-related thyroid disease as a potential, if still ill-defined, climate-sensitive health concern may guide future research and public health action. Fixing these issues will require concerted effort by ecological science, public health, and clinical medicine to define and mitigate any emergent threats that result from ongoing environmental change.

## Data Availability

No new data were created or analyzed in this study. Data sharing is not applicable to this article.
